# Prognostic Value of Preclinical Markers after Radiotherapy of Metastatic Spinal Cord Compression—An Additional Analysis of Patients from Two Prospective Trials

**DOI:** 10.3390/cancers14102547

**Published:** 2022-05-22

**Authors:** Dirk Rades, Jon Cacicedo, Darejan Lomidze, Ahmed Al-Salool, Barbara Segedin, Blaz Groselj, Steven E. Schild

**Affiliations:** 1 Department of Radiation Oncology, University of Lubeck, 23562 Lubeck, Germany; ahmed.al-salool@uksh.de; 2 Department of Radiation Oncology, Cruces University Hospital/Biocruces Health Research Institute, 48903 Barakaldo, Bizkaia, Spain; jon.cacicedofernandezbobadilla@osakidetza.eus; 3 Radiation Oncology Department, Tbilisi State Medical University and Ingorokva High Medical Technology University Clinic, Tbilisi 0177, Georgia; dlomidze@hotmail.com; 4 Department of Radiotherapy, Institute of Oncology Ljubljana, University of Ljubljana, 1000 Ljubljana, Slovenia; bsegedin@onko-i.si (B.S.); bgroselj@onko-i.si (B.G.); 5 Department of Radiation Oncology, Mayo Clinic, Scottsdale, AZ 85259, USA; sschild@mayo.edu

**Keywords:** metastatic spinal cord compression, radiotherapy, prospective trials, survival, prognostic factors, preclinical markers

## Abstract

**Simple Summary:**

For personalized treatment of metastatic spinal cord compression (MSCC), a patient’s remaining lifespan plays an important role, which can be estimated using prognostic factors. This study used data from 190 patients with poor or intermediate survival prognoses previously included in prospective trials to evaluate the prognostic role of preclinical markers including hemoglobin, neutrophil-to-lymphocyte ratio (NLR), platelet-to-lymphocyte ratio (PLR), lactate dehydrogenase (LDH), and c-reactive protein (CRP) plus clinical factors. On univariate analyses, NLR, LDH, CRP, and clinical factors tumor type, ambulatory status, and sphincter function were significantly associated with survival. On multivariate analysis, LDH, CRP, tumor type, and ambulatory status proved to be independent predictors of survival. In addition to clinical factors, preclinical markers may support physicians to estimate survival prognoses and contribute to the treatment personalization of patients with MSCC.

**Abstract:**

For optimal personalization of treatment for metastatic spinal cord compression (MSCC), the patient’s survival prognosis should be considered. Estimation of survival can be facilitated by prognostic factors. This study investigated the prognostic value of pre-treatment preclinical markers, namely hemoglobin, neutrophil-to-lymphocyte ratio (NLR), platelet-to-lymphocyte ratio (PLR), lactate dehydrogenase (LDH), and c-reactive protein (CRP), in 190 patients from two prospective trials who had poor or intermediate survival prognoses and were irradiated for MSCC with motor deficits. In addition, clinical factors including radiation regimen, age, gender, tumor type, interval from tumor diagnosis to MSCC, number of affected vertebrae, visceral metastases, other bone metastases, time developing motor deficits, ambulatory status, sensory function, and sphincter function were evaluated. On univariate analyses, NLR (*p* = 0.033), LDH (*p* < 0.001), CRP (*p* < 0.001), tumor type (*p* < 0.001), pre-radiotherapy ambulatory status (*p* < 0.001), and sphincter function (*p* = 0.011) were significant. In the subsequent Cox regression analysis, LDH (*p* = 0.007), CRP (*p* = 0.047), tumor type (*p* = 0.003), and ambulatory status (*p* = 0.010) maintained significance. In addition to clinical factors, preclinical markers may help in estimating the survival of patients irradiated for MSCC. Additional prospective trials are warranted.

## 1. Introduction

Metastatic spinal cord compression (MSCC) can occur in up to 10% of cancer patients during the course of their malignant disease [1]. Upfront surgery has been increasingly used, since 2005, when a randomized trial showed that decompressive surgery plus stabilization followed by radiotherapy was superior to radiotherapy alone in carefully selected patients [2]. Since considerably less than half of patients with MSCC meet the eligibility criteria of this trial, radiotherapy alone still is the most common treatment for MSCC [1,2]. Several radiation treatment schedules exist that include single-fraction with 8 or 10 Gy, multi-fraction short-course (e.g., 20 Gy in 5 fractions, 24 Gy in 6 fractions or 25 Gy in 5 fractions), and multi-fraction longer-course (e.g., 30 Gy in 10, 37.5 Gy in 15 or 40 Gy in 20 fractions) schedules [1,3,4]. Since each treatment session can be stressful for the patients, it is important that the overall treatment time is as short as possible without compromising care.

In two randomized trials, single-fraction radiotherapy (1 × 8 or 1 × 10 Gy) was not inferior to short-course treatment (20 Gy in 5 fractions) with respect to post-treatment ambulatory status in patients with very poor survival prognoses [5,6]. In another randomized trial that compared 20 Gy in 5 fractions to 30 Gy in 10 fractions in patients with poor to intermediate survival prognoses, motor function, ambulatory status, local progression-free survival (LPFS and overall survival (OS) were not significantly different [7]. No randomized trial has focused on patients with favorable survival prognoses treated with radiotherapy alone, since the majority of these patients receive upfront surgery [1,2]. However, in a prospective non-randomized trial, longer-course radiotherapy with 30–40 Gy in 10–20 fractions when compared to 1 × 8 Gy or 20 Gy in 5 fractions resulted in significantly better 1-year local control of MSCC [8]. Moreover, in 2011 a retrospective matched-pair study was presented that included only patients with (very) favorable prognoses receiving radiotherapy alone [9]. In this study, 191 patients irradiated with 30 Gy in 10 fractions were matched 1:1 to 191 patients receiving higher doses, namely 37.5 Gy in 15 fractions or 40 Gy in 20 fractions. Higher doses were associated with significantly better local control of MSCC (no in-field recurrence following radiotherapy), LPFS, and OS.

When considering these studies, patients with very poor survival prognoses appear appropriately treated with single-fraction radiotherapy, patients with poor to intermediate prognoses may receive multi-fraction short-course radiotherapy, and patients with favorable prognoses may benefit from longer-course programs, in case of very good prognoses with total doses >30 Gy [5,6,7,8,9]. Thus, when choosing an individualized radiation schedule, it is important to know the patient’s survival prognosis. To support physicians in this matter, prognostic factors of survival were identified and a validated survival score was developed [10,11,12]. However, the score was created from retrospective data and did not consider preclinical markers.

In addition to the prognostic value of pre-treatment hemoglobin levels for predicting survival, the role of other preclinical markers has been increasingly investigated in cancer patients with metastatic disease. This included, for example, the neutrophil-to-lymphocyte ratio (NLR), the platelet-to-lymphocyte ratio (PLR), the lactate dehydrogenase (LDH), and the C-reactive protein (CRP) [13,14,15,16,17,18,19,20,21,22,23,24,25,26,27,28,29,30,31]. For example, in a retrospective study of patients with metastatic prostate cancer, an elevated NLR was significantly associated with higher prostate-specific antigen levels [15]. In another retrospective study, a lower NLR predicted better survival in patients with metastatic or recurrent breast cancer [16]. A higher PLR was found to be a significant prognostic factor for survival in a retrospective cohort of patients with synchronous metastatic renal cell carcinoma [18]. Moreover, high LDH and CRP levels were significantly associated with worse survival in a retrospective series of patients with metastatic breast cancer [19]. Some studies investigated one or more of these factors in patients with bone metastases without symptomatic MSCC [20,21,22,23,24,25,26,27,28,29,30,31]. Lower hemoglobin levels and increased LDH levels were found to be associated with worse survival in studies of patients with bone metastases from prostate cancer or breast cancer [22,23,24,27,29,30]. Moreover, lower NLR and lower PLR were significantly associated with improved survival in a retrospective study of patients with bone metastases from different malignancies, most commonly from lung cancer or breast cancer [25]. The CRP was found to predict survival in patients receiving surgery for metastases of the long bones [31].

Except for one review of two retrospective studies with 39 melanoma patients that looked at hemoglobin and LDH, no study investigated the prognostic role of pre-treatment hemoglobin, NLR, PLR, LDH, and CRP in patients with symptomatic MSCC [13]. The present study evaluated these preclinical markers plus clinical factors for associations with survival in patients with MSCC from two prospective trials [7,32,33]. Preclinical markers may provide additional information regarding the survival prognoses of patients with MSCC and further improve personalization of their treatment.

## 2. Results

Median survival time in the entire cohort was 3.5 months. No significant difference was found between the two trials (*p* = 0.29). On univariate analyses (Table 1), NLR ≤ 5 (*p* = 0.033), LDH ≤ 250 U/L (*p* < 0.001), CRP ≤ 25 mg/L (*p* < 0.001), favorable tumor type (breast cancer and myeloma/lymphoma followed by prostate cancer, *p* < 0.001), being ambulatory prior to radiotherapy (*p* < 0.001), and normal sphincter function (*p* = 0.011) were significantly associated with survival.

In the subsequent Cox regression analysis, the preclinical markers LDH (risk ratio [RR] 1.81, *p* = 0.007) and CRP (RR 1.52, *p* = 0.047) proved to be independent predictors of survival. In addition, the clinical factors tumor type (RR 1.24, *p* = 0.003), and pre-radiotherapy ambulatory status (RR 1.79, *p* = 0.010) maintained significance. Results of the multivariate analysis including the 95% confidence intervals are shown in Table 2.

## 3. Discussion

The majority of patients with MSCC receive radiotherapy, either alone or following decompressive surgery [1]. Since upfront surgery is generally performed in carefully selected patients, the majority of patients with MSCC receive radiotherapy alone [1,2,3]. To avoid patients having to spend more time receiving radiotherapy than necessary, the number of treatment sessions should be kept as low as needed. The decision for a specific schedule is based on several factors including the patient’s remaining lifespan. Patients with a very limited lifespan may receive radiotherapy with a single fraction of 8 or 10 Gy. In a non-inferiority phase III trial, randomized patients received either 1 × 8 Gy (*n* = 345) or 20 Gy in 5 fractions (*n* = 341) [5]. A total of 342 patients were evaluable for the primary endpoint, ambulatory status after 8 weeks. Ambulatory rates at 8 weeks were 69.3% after 8 Gy and 72.7% after 20 Gy, respectively (*p* = 0.06 for non-inferiority). In another non-inferiority phase III trial, 73 patients were available for assessment of ambulatory rates at 5 weeks, which were 78% after 1 × 10 Gy and 65% after 20 Gy in 5 fractions, respectively (*p* = 0.34) [6].

Another phase III trial (*n* = 203) compared 20 Gy in 5 fractions to 30 Gy in 10 fractions in patients with poor to intermediate survival prognoses [7,10]. At 1 month following radiotherapy, improvement of motor function was found in 38.5% vs. 44.2% of patients, no further progression in 48.7% vs. 45.5% of patients, and deterioration in 12.8% vs. 10.4% of patients, respectively (*p* = 0.44). Ambulatory rates were 71.8% vs. 74.0% (*p* = 0.86). Six-month LPFS rates were 75.2% vs. 81.8% (*p* = 0.51), and 6-month OS rates 42.3% vs. 37.8% (*p* = 0.68). Thus, patients with poor to intermediate prognoses appear well treated with 20 Gy in 5 fractions.

In a prospective non-randomized trial of 265 patients, longer-course radiotherapy with 30–40 Gy in 10–20 fractions (*n* = 134) was compared to 1 × 8 Gy and 20 Gy in 5 fractions (*n* = 131) [8]. In those patients, in whom radiotherapy resulted in improvement or at least no further progression, 1-year local control rates of MSCC were 81% and 61%, respectively (*p* = 0.005). Since the risk of an in-field recurrence of MSCC increases with lifetime, local control is particularly important for longer-term survivors. Thus, patients with a remaining lifespan of more than 6 months should receive longer-course radiotherapy [8]. Patients with very favorable prognoses may even benefit from longer-course treatment with doses higher than 30 Gy. In a retrospective matched-pair study of 382 patients, 37.5 Gy in 15 fractions and 40 Gy in 20 fractions resulted in significantly better local control of MSCC and OS than 30 Gy in 10 fractions [9]. Therefore, for patients with very favorable prognoses who are not candidates for upfront surgery, longer-course radiotherapy with doses > 30 Gy appears appropriate.

When considering the results of these studies [5,6,7,8,9], it becomes obvious that it is very important to estimate a patient’s survival before developing an individual treatment plan. Estimation of a patient’s remaining lifespan is facilitated by using prognostic factors. Several clinical predictors of survival after radiotherapy for MSCC were already identified [10].

The prognostic role of pre-treatment preclinical markers such as hemoglobin, NLR, PLR, LDH, and CRP has not yet been sufficiently explored for patients with symptomatic MSCC. A few studies investigated one or more of these markers in patients with bone metastases [21,22,23,24,25,26,27,28,29,30,31]. In a retrospective study of 649 patients surgically treated for spinal metastases but not focusing on symptomatic MSCC, lower hemoglobin levels were independently associated with worse survival (hazard ratio = 0.92, *p* = 0. 009) [21]. In another retrospective study of 1901 patients with bone metastases from castration-resistant prostate cancer, hemoglobin levels >12.8 g/dL were associated with improved survival (*p* < 0.0001) [22]. Moreover, in a retrospective study of 435 breast cancer patients with bone metastases receiving bisphosphonates, hemoglobin levels > 12 g/dL were positively associated with survival at least in univariate analysis (*p* = 0.0066) [24]. In the study of Wedin et al., which was included in the systematic review of 39 patients with MSCC from melanoma, hemoglobin levels ≤ 11.5 g/dL had an unfavorable prognostic impact on survival [34]. In the present study, hemoglobin levels ≤ 11.5 g/dL showed a trend for an association with worse survival on univariate analysis. In addition to being a potential surrogate marker for more advanced disease, the negative impact of lower hemoglobin levels on survival can be explained by the fact that anemia results in worse oxygenation of the metastases responsible for spinal cord compression. Since the effect of radiotherapy is based on the induction of cytotoxic oxygen free radicals, impaired oxygenation of tumor cells decreases the antitumor effect of radiotherapy [35].

In the systematic review of 39 patients with MSCC from melanoma, worse survival was associated also with higher LDH levels [13]. This finding supports the results of the present study, where the LDH level was an independent prognostic factor inversely associated with survival. Higher LDH levels were associated with worse survival in other retrospective studies of patients with bone metastases from prostate cancer or breast cancer [22,23,24,27,29,30]. LDH is known as a key enzyme in many cells and tissues and is involved in the process of energy production [24]. Elevation of LDH occurs after injury of tissues, which can be caused also by cancer-related destruction of cells, and, therefore, is a marker of the activity of the malignant disease.

Moreover, the CRP was an independent predictor of survival in the present study. This preclinical factor has not been investigated in patients with MSCC before. However, CRP was found to be an independent predictor of 12-month survival in a prospective study of 159 patients who were surgically treated for metastases of the long bones [31]. This finding was explained by the fact that an elevated CRP is a biochemical marker of increased inflammatory activity.

In addition, in the univariate analyses of the present study, higher NLR was significantly associated with worse survival and higher PLR showed a trend. These findings were in line with the results of a previous retrospective study of 1012 patients with bone metastases from different malignancies [25]. In that study, 3-month survival rates were 61.3% in patients with high NLR compared to 84.0% in patients with low NLR (*p* < 0.001) and 55.6% in patients with high PLR compared to 75.8% in patients with low PLR (*p* < 0.001), respectively. These findings can be explained by the fact that NLR represents a component of an inflammatory response, which is an essential characteristic of cancer. One generally accepted hypothesis is that inflammation leads to increased production and release of neutrophils and decreased production of lymphocytes [25]. Thus, a greater NLR represents a more pronounced inflammatory activity.

The results of the previous and the present study suggest that preclinical markers may be helpful for predicting the survival of patients with MSCC. Several previous studies showed significant associations between one or more of the preclinical markers investigated in this study and survival in patients with bone metastases in general [13,21,22,23,24,25,26,27,28,29,30,31]. However, since these studies did not focus on patients with symptomatic MSCC, the results cannot be generalized to these patients. Therefore, the present study is important for this particular group of patients. However, when interpreting the results of this study, its limitations should be considered including the retrospective design, which bears the risk of hidden selection biases although only patients from prospective trials were included. Moreover, the five preclinical markers were not available in all patients, which may also have led to a hidden bias. Since the present study focused on patients with poor or intermediate survival prognoses according to an existing clinical survival score [11,12], in order to reduce the heterogeneity between the included trials, its results may not be applicable to patients with favorable prognoses. However, LDH and CRP, which were independent predictors of survival in this study, may contribute to the identification of patients with better prognoses and avoidance of undertreatment of these patients [11,12].

## 4. Materials and Methods

This additional study, which used data from patients from previous prospective trials, achieved approval from the ethics committee of the University of Lübeck (reference 21-478; approval of an amendment on 11 February 2022) [7,32,33]. Initially, it was planned to include data of 238 patients from one randomized phase III trial [7] and two prospective non-randomized phase II trials [32,34,35]. The cut-off values for the five investigated preclinical markers were chosen based on the median values from this cohort. However, since a favorable survival prognosis was a criterion for inclusion in one of the phase II trials [36], it was decided to exclude the patients from this trial in order to reduce heterogeneity. Moreover, since the majority of patients with MSCC have poor or intermediate survival prognoses [11,12] and the phase III trial [7] focused on these groups, the six patients with favorable prognoses from the remaining phase II trial [34,35] were also not included in the present study. The major goal of this procedure was a reduction of the heterogeneity between both trials. Indeed, the survival between the trials was not significantly different (*p* = 0.29, log-rank test). Finally, 190 patients treated with radiotherapy alone (without upfront surgery) after presentation to a surgeon were included in the current retrospective study. These patients had motor deficits of the lower extremities due to MSCC of the thoracic or lumbar spine existing for no longer than 30 days. They received fractionated radiotherapy with conventional radiotherapy (*n* = 165) or high-precision techniques (*n* = 25) including volumetric modulated arc therapy (VMAT) and intensity-modulated radiation therapy (IMRT) between 2010 and 2018. Further details of radiotherapy were previously reported [7,34,35]. In both trials, follow-up periods were 6 months. For the current study, additional follow-up data were obtained from patient files and from telephone interviews with patients, relatives, and treating physicians.

The major goal of this study was the evaluation of potential associations between five preclinical markers and survival. The markers were assessed up to 2 weeks prior to radiotherapy and included hemoglobin level (≤11.5 vs. >11.5 g/dL), NLR (≤5 vs. >5), PLR (≤250 vs. >250), LDH (≤250 vs. >250 U/L), and CRP (≤25 vs. >25 mg/L). Patients with chronic anemia or current infections were not excluded. In addition to these preclinical markers, clinical factors including radiotherapy regimen (short-course vs. longer-course), age (≤68 vs. >68 years, media*n* = 68.5 years, gender (female vs. male), primary tumor type (breast cancer vs. prostate cancer vs. myeloma/lymphoma vs. lung cancer vs. other tumors), interval between tumor diagnosis and MSCC (≤6 vs. >6 months, media*n* = 6 months), number of vertebrae affected by MSCC (1–2 vs. ≥3, media*n* = 2), visceral metastases at start of radiotherapy (no vs. yes), other bone metastases at start of radiotherapy (no vs. yes), time developing motor deficits prior to radiotherapy (0–7 vs. 8–14 vs. >14 days), ambulatory status prior to radiotherapy (not ambulatory vs. ambulatory), sensory function (impaired vs. normal), and sphincter function (impaired vs. normal). Short course radiotherapy was 5 × 4 Gy or 5 × 5 Gy over 1 week, and longer-course radiotherapy included 10 × 3 Gy, 14–15 × 2.5 Gy, or 20 × 2 Gy over 2–4 weeks. The distributions of all investigated factors are summarized in Table 3. The patients’ performance status was not included, because the Eastern Cooperative Oncology Group (ECOG) performance score and ambulatory status are confounding variables. Ambulatory patients generally had ECOG scores of 0–2, and non-ambulatory patients had ECOG scores of 3–4.

Survival was calculated from the last day of radiotherapy. Patients were followed until death or for at least 3 months. Univariate analyses were performed with the Kaplan-Meier method and the log-rank test for comparing the Kaplan-Meier curves (BlueSky Statistics 10 GA, BlueSky Statistics LLC, Chicago, IL, USA). Factors that were significant in univariate analysis (*p* < 0.05) were included in a multivariate analysis performed with the Cox regression model. Again, *p*-values of <0.05 were considered significant.

## 5. Conclusions

In conclusion, preclinical markers were identified in addition to clinical factors that may support treating physicians in estimating the survival of patients irradiated for MSCC. This data may help improve treatment personalization for these patients. Since the study focused on patients with poor or intermediate survival prognoses (according to a clinical survival score), the results may not be applicable to patients with favorable prognoses. Moreover, the retrospective nature of the present study needs to be considered when interpreting its results. Although the data were obtained from prospective trials, the risk of a hidden selection bias remains. Additional prospective trials are warranted to better define the prognostic role of preclinical markers for survival in patients with MSCC.

## Figures and Tables

**Table 1:** Univariate analyses: Survival rates (in %) at 3, 6, 9, and 12 months following radiotherapy.

Characteristic	At 3 mos.	At 6 mos.	At 9 mos.	At 12 mos.	*p*-Value
Pre-RT hemoglobin level					0.12
≤11.5 g/dL (*n* = 97)	46	38	28	25	
>11.5 g/dL (*n* = 92)	54	42	37	37	
Neutrophil-to-lymphocyte ratio					**0.033**
≤5 (*n* = 77)	61	50	41	37	
>5 (*n* = 98)	40	30	26	26	
Platelet-to-lymphocyte ratio					0.14
≤250 (*n* = 81)	57	47	40	37	
>250 (*n* = 94)	43	32	25	25	
Lactate dehydrogenase					**<0.001**
≤250 (*n* = 82)	68	55	44	44	
>250 (*n* = 91)	32	24	18	16	
C-reactive protein					**<0.001**
≤25 (*n* = 75	65	49	42	38	
>25 (*n* = 92)	36	32	23	23	
Radiotherapy regimen					0.45
Short-course RT (*n* = 110)	48	42	33	33	
Longer-course RT (*n* = 80)	53	37	31	28	
Age					0.29
≤68 years (*n* = 95)	45	35	31	31	
>68 years (*n* = 95)	55	45	34	30	
Gender					0.21
Female (*n* = 78)	56	47	42	40	
Male (*n* = 112)	46	35	26	25	
Type of primary tumor					**<0.001**
Breast cancer (*n* = 32)	78	69	60	60	
Prostate cancer (*n* = 24)	63	54	48	41	
Myeloma/lymphoma (*n* = 16)	69	69	69	69	
Lung cancer (*n* = 56)	36	23	13	13	
Other tumor types (*n* = 62)	39	25	22	20	
Interval tumor diagnosis to MSCC					0.14
≤6 months (*n* = 102)	52	42	36	36	
>6 months (*n* = 88)	48	37	28	24	
Number of affected vertebrae					0.067
1–2 (*n* = 102)	55	43	36	32	
≥3 (*n* = 88)	44	36	29	29	
Visceral metastases					0.41
No (*n* = 48)	63	50	39	35	
Yes (*n* = 142)	46	37	30	29	
Other bone metastases					0.95
No (*n* = 23)	57	30	30	30	
Yes (*n* = 167)	49	41	33	31	
Time developing motor deficits					0.44
0–7 days (*n* = 82)	49	39	32	30	
8–14 days (*n* = 50)	54	42	39	39	
>14 days (*n* = 58)	48	40	28	25	
Pre-RT ambulatory status					**<0.001**
Not Ambulatory (*n* = 81)	35	28	19	16	
Ambulatory (*n* = 109)	61	49	42	40	
Pre-RT Sensory function					0.95
Impaired (*n* = 94)	52	39	33	31	
Normal (*n* = 93)	49	42	33	32	
Pre-RT Sphincter function					**0.011**
Impaired (*n* = 49)	39	29	21	21	
Normal (*n* = 141)	54	44	36	34	

Pre-RT: prior to radiotherapy; MSCC: metastatic spinal cord compression**;** bold *p*-values are significant.

**Table 2:** Results of the multivariate analysis of survival.

Characteristic	Risk Ratio	95% Confidence Interval	*p*-Value
Neutrophil-to-lymphocyte ratio	1.08	0.73–1.60	0.69
Lactate dehydrogenase	1.81	1.17–2.78	**0.007**
C-reactive protein	1.52	1.00–2.31	**0.047**
Type of primary tumor	1.24	1.07–1.43	**0.003**
Pre-RT ambulatory status	1.79	1.15–2.79	**0.010**
Pre-RT Sphincter function	1.00	0.61–1.63	>0.99

Pre-RT: prior to radiotherapy; bold *p*-values are significant.

**Table 3:** Characteristics investigated for associations with survival.

Characteristic	Number of Patients	Proportion (%)
Pre-RT hemoglobin level		
≤11.5 g/dL	97	51
>11.5 g/dL	92	48
Unknown	1	1
Neutrophil-to-lymphocyte ratio		
≤5	77	41
>5	98	52
Unknown	15	8
Platelet-to-lymphocyte ratio		
≤250	81	43
>250	94	49
Unknown	15	8
Lactate dehydrogenase		
≤250	82	43
>250	91	48
Unknown	17	9
C-reactive protein		
≤25	75	39
>25	92	48
Unknown	23	12
Radiotherapy regimen		
Short-course RT	110	58
Longer-course RT	80	42
Age		
≤68 years	95	50
>68 years	95	50
Gender		
Female	78	41
Male	112	59
Type of primary tumor		
Breast cancer	32	17
Prostate cancer	24	13
Myeloma/lymphoma	16	8
Lung cancer	56	29
Other tumor types	62	33
Interval tumor diagnosis to MSCC		
≤6 months	102	54
>6 months	88	46
Number of affected vertebrae		
1–2	102	54
≥3	88	46
Visceral metastases		
No	48	2
Yes	142	75
Other bone metastases		
No	23	12
Yes	167	
Time developing motor deficits pre-RT		
0–7 days	82	43
8–14 days	50	26
>14 days	58	31
Pre-RT ambulatory status		
Not Ambulatory	81	43
Ambulatory	109	57
Pre-RT Sensory function		
Impaired	94	49
Normal	93	49
Unknown	3	2
Pre-RT Sphincter function		
Impaired	49	26
Normal	141	74

Pre-RT: prior to radiotherapy; MSCC: metastatic spinal cord compression.

## Data Availability

Data of two prospective trials (NCT03070431, NCT02189473) are available at clinicaltrials.gov (accessed on 21 April 2022). Otherwise, the data analyzed for this paper cannot be shared due to data protection regulations. According to the ethics committee, only evaluation of anonymized data is allowed for this study.
